# Draft Genome Sequence of *Blastomonas* sp. Strain CACIA 14H2, a Heterotrophic Bacterium Associated with Cyanobacteria

**DOI:** 10.1128/genomeA.01200-13

**Published:** 2014-01-16

**Authors:** Alex Ranieri Jerônimo Lima, Andrei Santos Siqueira, Bruno Garcia Simões dos Santos, Fábio Daniel Florêncio da Silva, Clayton Pereira Lima, Jedson Ferreira Cardoso, João Lídio S. G. Vianez-Júnior, Márcio Roberto Teixeira Nunes, Evonnildo Costa Gonçalves

**Affiliations:** Laboratório de Tecnologia Biomolecular, Instituto de Ciências Biológicas (ICB), Universidade Federal do Pará (UFPA), Belém, Pará, Brazila; Centro de Inovações Tecnológicas (CIT), Instituto Evandro Chagas (IEC), Ananindeua, Pará, Brazilb

## Abstract

With the new methods for assembling sequence data from metagenomic samples, the genomic study of heterotrophic bacterium-cyanobacterium associations can now be improved. In this work, the draft genome sequence of *Blastomonas* sp. strain CACIA 14H2, obtained from a nonaxenic culture of a *Cyanobium* sp., is presented.

## GENOME ANNOUNCEMENT

The development of new methods of assembling sequencing data from metagenomic samples (1, 2) means it has become possible to address via genome sequences the unique features shared by heterotrophic bacterium-cyanobacterium associations (3) that are capable of producing several compounds of biotechnological interest (4, 5). Thus, we obtained the draft genome of *Blastomonas* sp. strain CACIA 14H2 from the sequencing of a nonaxenic culture of cyanobacteria. The genus *Blastomonas* is characterized by rod-shaped Gram-negative bacteria that produce bacteriochlorophyll α under aerobic conditions (6, 7). So far, public databases have presented only one draft genome sequence for the genus (8).

The cyanobacterial isolate was obtained from a water sample collected in December 2010 from the Tucuruí hydroelectric dam (3°49′55″S, 49°38′50″W), Pará, Brazil. DNA samples were obtained from *Cyanobium* sp. strain CACIA 14 cultured 6 months apart. The two nonpaired libraries (9) were sequenced using the GS FLX 454 sequencer (Roche Life Science), resulting in 660,228 (~255 Gbp) and 815,325 (~357 Gbp) reads for the first and second runs, respectively. Both datasets were assembled separately with Newbler 2.6 (minimum read size, 45 bp; minimum overlap, 40 bp; minimum overlap identity, 90%). These analyses generated 3,654 and 3,256 contigs >1 kbp in length, with N_50_ values of 2,149 bp and 37,998 bp. The assembled contigs were identified and separated using a metagenomic assembling pipeline (1) for each putative organism. The assembled contigs from the second run were used to determine the genome coverage. The draft genome of *Blastomonas* sp. CACIA 14H2 contains 72 contigs (4,067,409 bp), ranging from 5,698 to 260,673 bp; the average coverage was estimated to be 25×, the N_50_ is 89,796, and the G+C content is 65.24%. The pipeline uses 107 hidden Markov models for essential genes present in a single copy in 95% of all bacteria. The draft contains 106 of these genes, with PF00750 in duplicate, which normally occurs this way. The structural annotation was performed with the PGAP tool, available on the NCBI website (10), resulting in 3,787 annotated coding sequences (CDSs) and 42 tRNA genes. The rRNA clusters were predicted by the RNAmmer tool (11). The 16S rRNA predicted is 99% similar to the relative sequence of *Blastomonas natatoria* strain DSM 3183, while the *rpoB* gene is 97% similar to the relative sequence of *Blastomonas* sp. strain AAP53.

The information contained in the draft genome of *Blastomonas* sp. CACIA 14H2 presented here may be useful for elucidating attributes important to heterotrophic bacterium-cyanobacterium associations.

### Nucleotide sequence accession numbers.

This whole-genome shotgun project has been deposited at DDBJ/EMBL/GenBank under the accession no. AYSC00000000. The version described in this paper is version AYSC01000000.
